# Supplementary material for: Seed- and Soil-Dependent Differences in Murine Breast Tumor Microenvironments Dictate Anti-PD-L1 IgG Delivery and Therapeutic Efficacy
Source: Pharmaceutics. 2021 Apr 10;13(4):530. doi: 10.3390/pharmaceutics13040530 (PMC8069710; doi:10.3390/pharmaceutics13040530)
Supplement: Supplementary file 1 [file pharmaceutics-13-00530-s001.pdf]

pharmaceutics

MDPI

Supplementary Materials: Seed- and Soil-Dependent Differences in Murine Breast Tumor Microenvironments Dictate Anti-PD-L1 IgG Delivery and Therapeutic Efficacy

Yan Ting Liu, Shreya Goel, Megumi Kai, Jose Alberto Moran Guerrero, Thao Nguyen, Junhua Mai, Haifa Shen, Arturas Ziemys and Kenji Yokoi

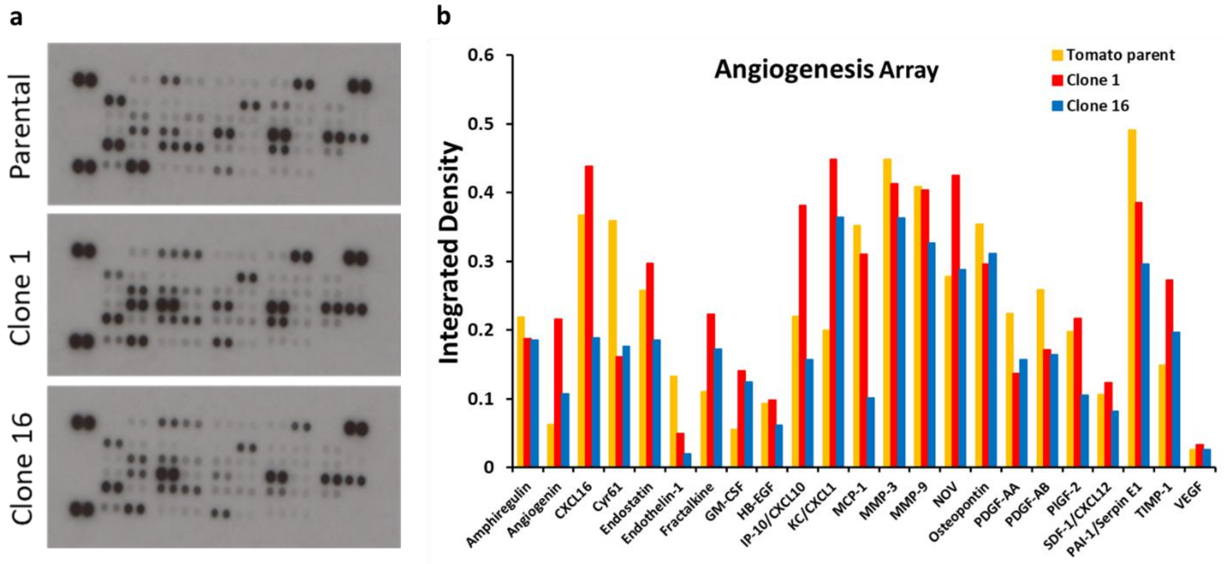

Figure S1. In vitro quantification of angiogenesis proteins. Angiogenesis assay was performed to quantify the amount each protein present in parental, Clone 1 and Clone 16 cell culture supernates.

Publisher's Note: MDPI stays neutral with regard to jurisdictional claims in published maps and institutional affiliations.

Copyright: © 2020 by the authors. Submitted for possible open access publication under the terms and conditions of the Creative Commons Attribution (CC BY) license (http://creativecommons.org/licenses/by/4.0/).

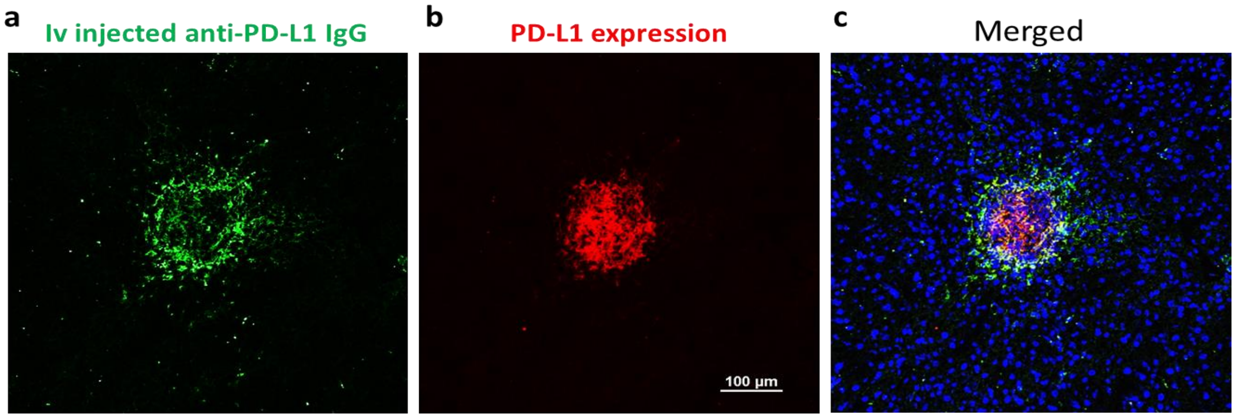

Figure 2. Limited distribution of iv injected fluorescently labeled anti-PD-L1 IgG into liver metastasis. Delivery of fluorescently labeled anti-PD-L1 IgG into liver metastasis was limited to the border of liver metastasis (**a**) although entire tumor expressed PD-L1 (**b**). Merged image is shown in (**c**).

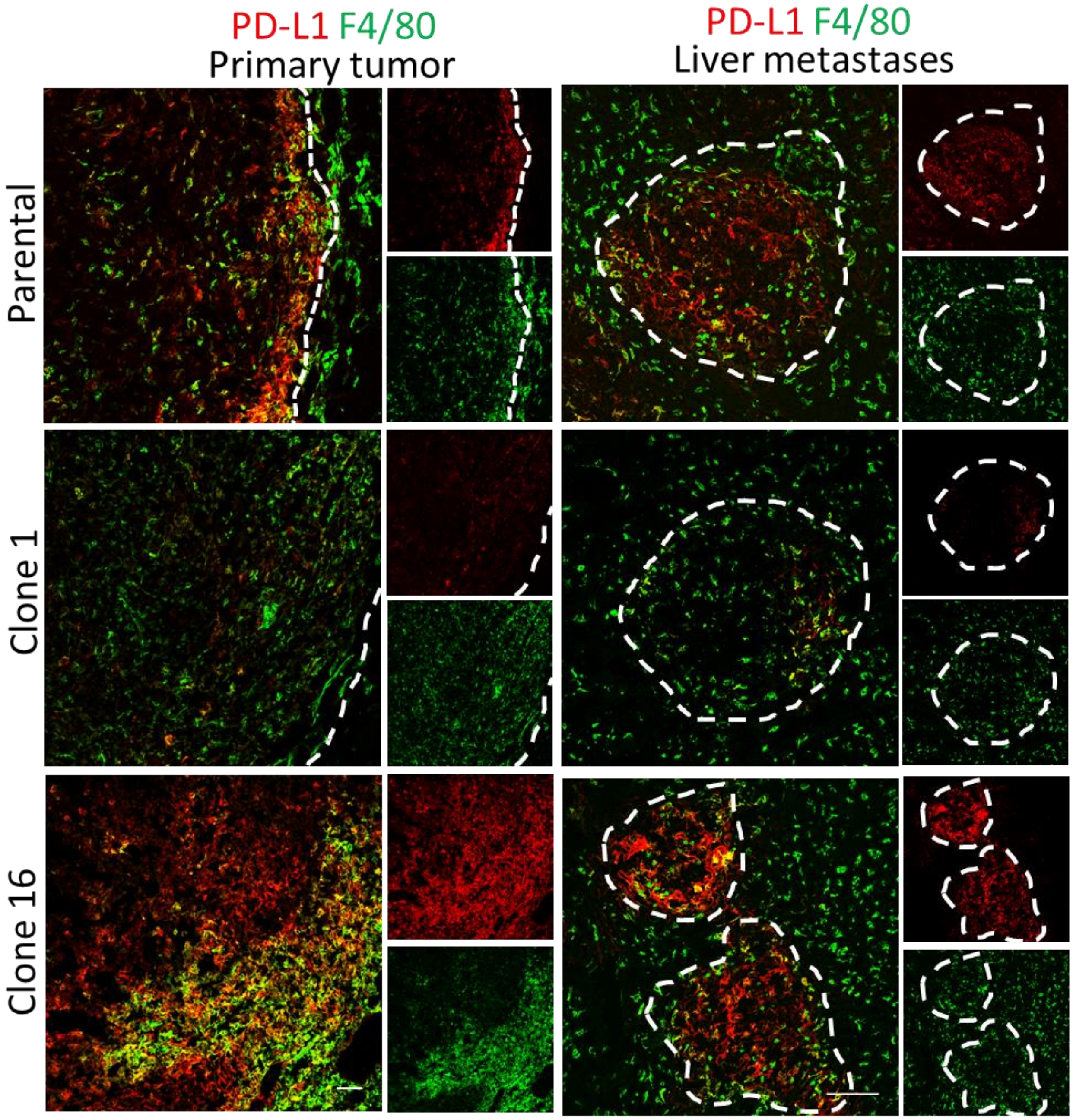

Figure 3. Verification of immune maker that express PD-L1 by immunohistochemical imaging of primary tumor and liver metastases. Immunofluorescence imaging of PD-L1 expression (red) and F4/80 (green) in parental-, Clone-1-, Clone-16-derived primary tumors and liver metastases. Scale bar, 100μm.

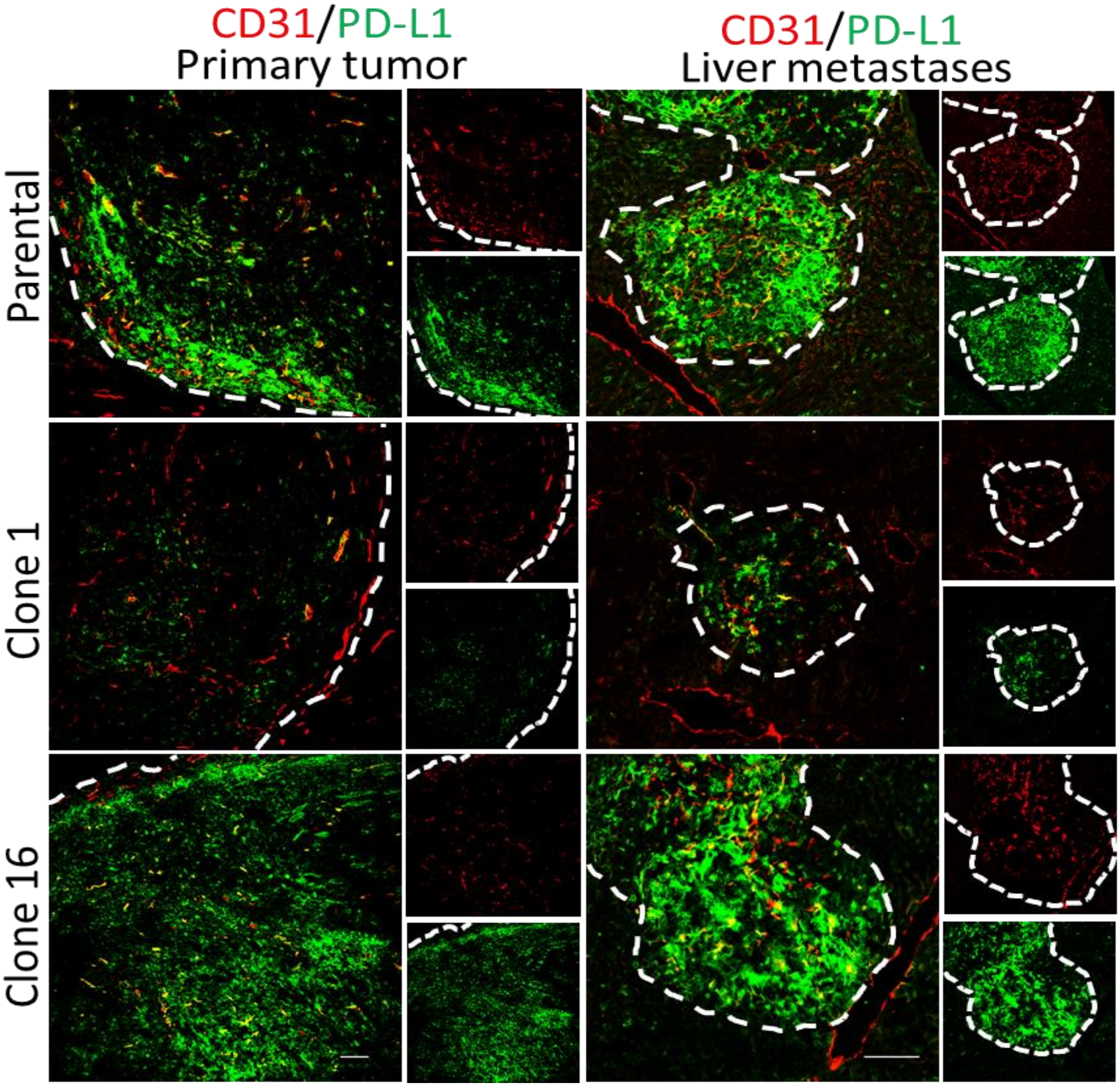

Figure 4. Verification of endothelial cells (CD31) that express PD-L1 by immunohistochemical imaging of primary tumor and liver metastases. Immunofluorescence imaging of PD-L1 expression (green) and CD31(red) in parental-, Clone-1-, Clone 16-derived primary tumors and liver metastases. Scale bar, 100μm.
